# Evaluating home-based personalised virtual reality physiotherapy rehabilitation compared with usual care in the treatment of pain for people with knee osteoarthritis: protocol for a randomised feasibility study

**DOI:** 10.1136/bmjopen-2025-102994

**Published:** 2025-10-21

**Authors:** Mohammad Al-Amri, Samuel Bird, Denise-Teodora Nistor, Judith White, Kate Button, Martin Warner, David Walsh, Dione Shorten, Rose Evans

**Affiliations:** 1 Cardiff University School of Healthcare Studies, Cardiff, UK; 2 Biomedical Engineering Department, The Hashemite University, Zarqa, Jordan; 3 CEDAR (Centre for Healthcare Evaluation, Device Assessment, and Research), Cardiff and Vale University Health Board, Cardiff, UK; 4 School of Health Sciences, University of Southampton Faculty of Environmental and Life Sciences, Southampton, UK; 5 Academic Rheumatology, University of Nottingham Faculty of Medicine and Health Sciences, Nottingham, UK; 6 Cardiff and Vale University Health Board, Cardiff, UK; 7 Physiotherapy, Cardiff and Vale University Health Board, Cardiff, UK

**Keywords:** Clinical Trial, Knee, Pain management, Exercise, Feasibility Studies, Rehabilitation medicine

## Abstract

**Introduction:**

Home-based physiotherapy is a current approach to manage knee osteoarthritis (OA). However, adherence to physiotherapy is poor. Non-immersive virtual reality (VR) has shown promise in improving self-efficacy and adherence in other clinical conditions. A non-immersive VR-based home physiotherapy system named Sensor-based Physiotherapy Intervention with Virtual Reality (SPIN-VR) for knee OA has been developed, integrating physiotherapy exercises into engaging games that adjust in difficulty based on real-time performance. This approach aims to enhance exercise adherence by making physiotherapy more enjoyable and personalised. To evaluate the feasibility of this intervention, a randomised controlled trial is being conducted.

**Method and analysis:**

This single-centre, open-label, randomised controlled feasibility trial will evaluate the SPIN-VR system over 12 weeks compared with usual care physiotherapy for knee OA. 50 participants will be randomly assigned to either the SPIN-VR or usual care group, with follow-ups at 12 and 24 weeks post randomisation. The primary outcomes will be a description of feasibility of recruitment, patient willingness to be randomised, the completeness of outcome measures and patient adherence to the intervention. Secondary outcomes include evaluations of muscle strength, endurance, aerobic capacity, exercise technique, central pain processing and self-reported pain mechanisms and moderators. Participants in the intervention arm will be interviewed after 12 weeks to capture their experience in using the VR system.

**Ethics and dissemination:**

This protocol was approved by the Wales Research Ethics Committee 3. Research findings will be disseminated in open-access peer-reviewed journals, to researchers and health professionals through conference presentations, to patients and the public by organising webinars and a seminar.

**Trial registration number:**

NCT06639867.

**STRENGTHS AND LIMITATIONS OF THIS STUDY**

- This study uses a comprehensive set of quantitative outcomes and patient-reported outcomes measures to evaluate feasibility, acceptability and potential mechanisms of effect.
- The inclusion of qualitative interviews allows further evidence and information about the feasibility of the study and intervention to be gathered.
- The main limitation of this study is the lack of blinding in the collection of follow-up data. It also lacks a power calculation to examine efficacy.
- Another limitation is that the standard care arm of the study is not controlled for. Any future studies should compare the intervention against a similar intervention, or a more controlled standard care treatment.

## Introduction

 Osteoarthritis (OA) is a costly major worldwide challenge, impairing function and quality of life. It is one of the leading causes of musculoskeletal pain and disability worldwide,^1^ mainly affecting the knees and hips. The global prevalence of knee OA in 2020 was approximately 7.6% of the global population^2^ and, with an ageing population, the projected rate of years lived with disability is estimated to be 118.5 per 100 000.^3^ Persistent pain is the dominant symptom and can be associated with widespread sensitisation, which may impair neuromuscular control.^4^ OA pain fluctuates, often with intermittent and severe flares from which both symptomatic and functional recovery might be incomplete. Ultimately, pain impairs the differential control of muscles around the painful area, leading to a loss of functional independence and a profound reduction in physical activity, quality of life and mental well-being.^5 6^

Physiotherapy-based exercise is a key non-pharmacological intervention for OA pain management.^7 8^ It offers a safer, low-cost alternative to surgery and long-term medication.^9^,^11^ Exercise reduces pain through multiple mechanisms, including activating endogenous pain inhibition (exercise-induced hypoalgesia),^12^,^14^ improving muscle perfusion^15 16^ and enhancing joint stability during daily activities.^17 18^ However, exercise benefits diminish without continued adherence. A major challenge in translating clinical trial efficacy into real-world outcomes is poor long-term compliance. Prolonged treatment is essential for sustained pain relief and functional improvements.^19^ Patients need skills and motivation to maintain exercises after clinical support ends, as delayed intervention can compromise future treatments like arthroplasty.^20^

Home-based physiotherapy is a promising solution, accommodating individual needs, time constraints and preferences. Studies suggest it can be as effective as clinic-based therapy,^21^,^24^ but adherence remains a concern.^25 26^ Effective home programmes must foster self-efficacy, ensuring exercises are relevant, enjoyable and aligned with patient goals. A Cochrane review found that people are more likely to sustain exercise when it is meaningful and enjoyable,^19^ suggesting that personalised, motivational exercise plans are crucial for long-term success.^19 27 28^

A potential innovative approach to improve physiotherapy is the inclusion of virtual reality (VR) technology. This technology has shown promise in rehabilitating conditions such as stroke and Parkinson’s disease.^29^,^31^ Moreover, a real-time VR feedback game has enabled people with OA to modify their squatting techniques based on targeted feedback.^32 33^ To personalise physiotherapy and allow patients to perform exercises in a controlled and engaging setting, Cardiff University’s Sensor Physiotherapy Intervention (SPIN) Research Group has developed a sensor-based VR physiotherapy intervention following Medical Research Council guidelines.^34^ Wearable and VR technologies can transform home-based physiotherapy for individuals with OA by enhancing adherence, ensuring correct technique and providing instant feedback of pain relief. This VR system integrates physiotherapy principles, movement science, intelligent algorithms and body-worn inertial measurement units (IMUs) to offer real-time and offline feedback on movement quality. It supports the assessment of key biomechanical parameters using data captured from IMUs. Certain parameters, such as joint angle attainment and movement consistency, are used in real time to guide adaptive gameplay. Other parameters, including estimation of squat depth, maximum joint angles, range of motion, movement symmetry and, depending on the sensor configuration, postural sway, are analysed offline to enable a more comprehensive evaluation.

The system is structured around three key components:

1. Monitoring performance.
2. Exercise goal setting.
3. Exercise progressions.

A built-in algorithm personalises the VR games, adjusting scenarios in real-time based on user progression and exercise quality. Adaptations rely on a composite score that integrates pain severity and movement patterns. When predefined thresholds are reached, task complexity increases, introducing dual-task challenges to maintain patient engagement and motivation throughout their physiotherapy journey. The objective of this feasibility study is to evaluate the practicality of implementing the VR home physiotherapy (SPIN-VR) programme for patient use in a home setting and to assess the feasibility of conducting a larger randomised controlled trial (RCT) to determine the clinical efficacy of SPIN-VR in comparison to usual physiotherapy care provided by the National Health Service (NHS).

## Methods and analysis

### Study design

This is a single-centre, open-label, randomised controlled feasibility trial of a 12-week SPIN-VR programme with a 24-week post-randomisation follow-up. 50 people diagnosed with knee OA who fulfil National Institute for Health and Care Excellence (NICE) and American College of Radiology (ACR) criteria for a knee OA diagnosis^6 35^ will be randomly assigned in equal numbers to either an SPIN-VR programme or to receive usual care physiotherapy.

The study is planned to run from August 2024 to the end of June 2026 when the last patient follow-up is expected.

The study aims to assess the following feasibility outcomes.

### Primary outcome measures

1. Feasibility of recruitment, measured by enrolling 4 patients per month.
2. Completeness of outcome measures, measured by the number (%) of each questionnaire and mechanistic outcome completed at 12 weeks and 24 weeks post randomisation.
3. Fidelity of healthcare professionals delivering intervention using treatment logs and face-to-face contact, and observation of two assessments and training sessions for setting up patients with knee OA with the intervention.
4. Acceptability of intervention and trial procedures through interviews with patients and staff about expectations and experience of the intervention, and barriers and facilitators to trial participation.
5. Adverse events (AEs) through treatment logs and patient interviews will be used to find issues related to knee symptoms or muscle soreness and falls, and motion sickness, plus any unexpected AEs.
6. Adherence to the intervention by the number of times and date/time of when patients logged in to the VR games and number of physiotherapy follow-up consultations.

### Secondary outcome measures

1. Evaluate the processes for exercise mechanism of action at improving pain outcomes through a variety of measurements relating to muscle strength and endurance, aerobic capacity, exercise technique, central pain processing, and self-reported pain outcomes and moderators.
2. Assess intervention to treat knee OA using the Outcome Measures in Rheumatology—Osteoarthritis Research Society International (OMERACT-OARSI) core domain set. A variety of patient-reported outcome measures. Pain sensitisation by algometer. Dynamic balance using a step test.

### Participants

The target population is adults with knee OA who fulfil NICE and ACR criteria for knee OA diagnosis^36 37^ and who have been referred to a physiotherapy clinic.

#### Inclusion criteria

1. Adults aged 45 years or older.
2. Clinical diagnosis of knee OA.
3. Referred for physiotherapy for clinically diagnosed knee OA pain.
4. Activity-related joint pain.
5. Self-reported knee pain on most days for the past 3 months.
6. Average pain severity in the past week of 4 or greater on a 10-point Numeric Rating Scale.
7. Able to understand written and spoken English.
8. Able to provide written informed consent.

#### Exclusion criteria

Where the knee is not identified by the participant as the main source of pain (eg, comorbid painful conditions, widespread pain).

1. Contraindication to exercise.
2. Pain caused by malignancy, fractures or inflammatory arthritis.
3. Has received surgery for their knee pain in the last 12 months, or had previous knee arthroplasty on the affected knee.
4. Has commenced another new treatment for knee pain, other than the trial interventions, during the preceding 12 weeks.
5. Unable to walk without a walking aid.
6. Unable or unwilling to engage in either active or control intervention.

### Trial assessments

**Figure 1:**
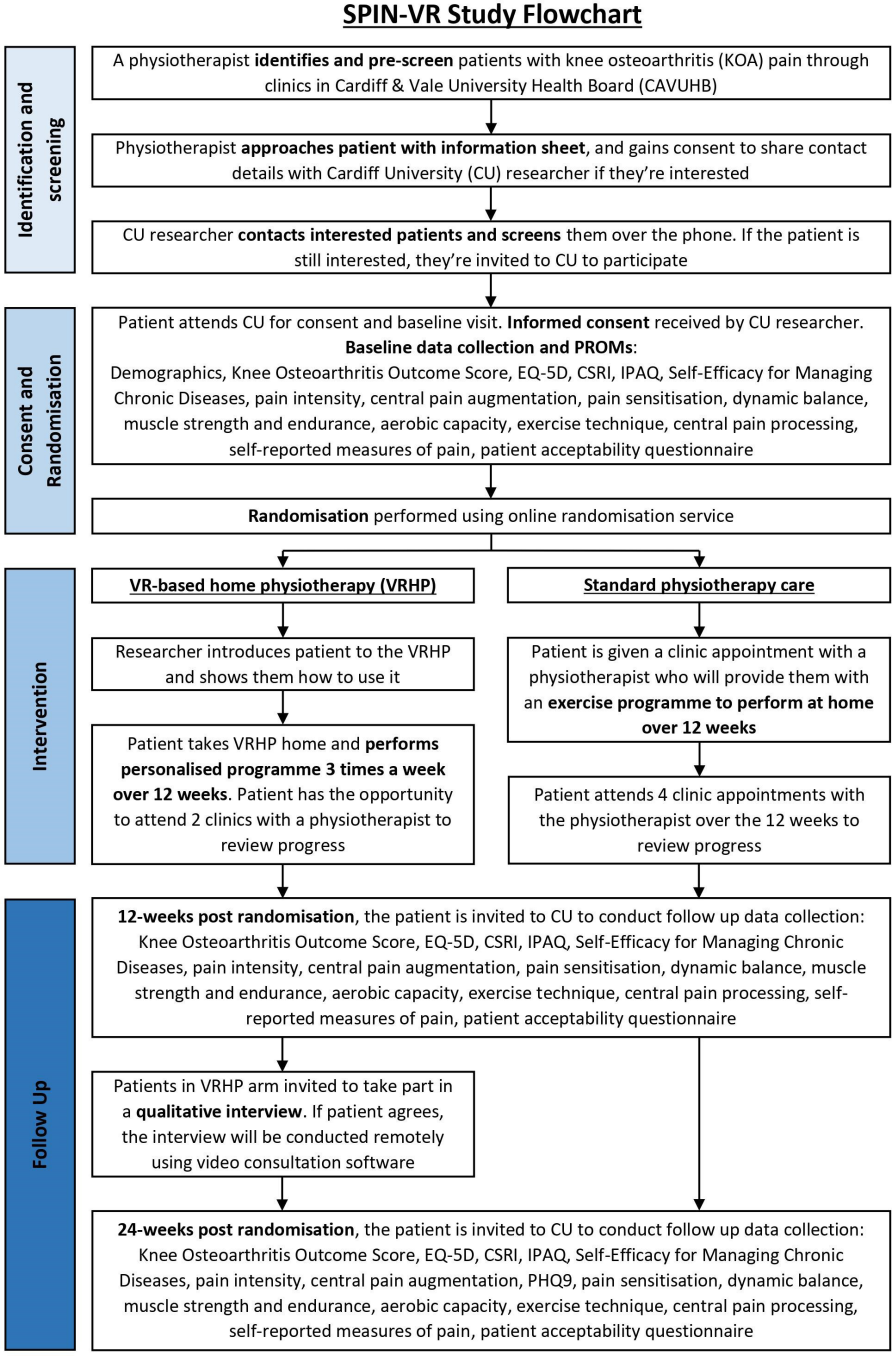
Flow diagram of patient study pathway of recruitment and follow-up. CSRI, Client Service Receipt Inventory; IPAQ, International Physical Activity Questionnaire; PHQ9, Patient Health Questionnaire-9; SPIN-VR, Sensor-based Physiotherapy Intervention with Virtual Reality.

An assessment will be carried out at the baseline and consent visit with a researcher. During the baseline visit, the research team will collect demographic data. Patients will have two in-person follow-up assessments with the Cardiff University (CU) research team, at 12 weeks post randomisation, which will occur at the end of their SPIN-VR programme, and another at 24 weeks post randomisation. A flowchart of the patient pathway is presented in figure 1.

The following assessments will be conducted at both baseline and follow-up visits:

1. Pain sensitisation, conducted using algometer.
2. Dynamic balance, using a validated step test by asking participants to maintain balance on the study leg, while stepping the contralateral leg on and off a 15 cm step as many times as possible in 15 s without any weight transfer to the stepping leg. A higher number of steps indicates a better outcome.
3. Evaluation of exercise mechanisms of action:
  - Muscle strength and endurance: 30 s time sit to stand test. A faster time indicates a better outcome.^38^
  - Aerobic capacity: timed 6 m walk test. A faster time indicates a better outcome.^39^
  - Exercise technique: throughout exercises by evaluating key biomechanical parameters for each exercise and compared with lab-based data collected on healthy subjects, for example, centre of mass motion.
  - Central pain processing: quantitative sensory testing modality of pressure pain detection thresholds local and distant from the index knee.^40^
  - Self-report measures of pain mechanisms and moderators: activity, medication use, psychological well-being.^41^

Participants will be invited to complete a series of patient-reported outcomes measures (PROMs). These are:

1. Knee Osteoarthritis Outcome Score (pain, symptoms, activities of daily living, function in sport and recreation, and knee-related quality of life). A higher score indicates a better outcome.^42^
2. EQ-5D-5L Questionnaire (overall health status).^43^
3. Client Service Receipt Inventory (healthcare utilisation, medication use, service receipt for arthritis).^44^
4. International Physical Activity Questionnaire^45^ (physical activity levels).
5. Self-Efficacy for Managing Chronic Diseases 6-Item Scale (confidence in managing chronic disease) A higher score indicates a better outcome.^46^
6. Central Aspects of Pain (constructs associated with pain across musculoskeletal conditions).^47^
7. Pain intensity before and after exercise. A lower score indicates a better outcome.
8. A patient acceptability questionnaire on the SPIN-VR intervention.

#### Qualitative interviews

Following recruitment, patient interviews will be conducted with a sample of 15 participants after the 12-week intervention. The purpose of these interviews is to gather valuable insights and perspectives regarding the study and the intervention. Topics covered will include the participants’ acceptance of the technology, factors influencing their participation in the trial, any unexpected benefits or adverse effects experienced, as well as their experiences with pain and any impact on their overall function and exercise. Interviews will last between 30 and 45 min. The audio files will be transcribed verbatim by a professional transcription company. The data will be analysed using thematic analysis and NVivo software will be used to manage the data analysis.

### Intervention

The intervention is SPIN-VR that consists of five biomechanically validated Movella Dot IMUs (Movella), which are positioned on the upper and lower limbs in accordance with the movement requirements of each virtual exercise. Sensor placement is task specific to ensure accurate motion capture and feedback during exercise performance. The games will be played on a laptop computer that can be connected to a TV to enable a larger view of the gaming environment. Connecting it to a TV is optional. Each participant will be provided with a unique login to the VR software, which will allow the research team to track the regularity of use and progress of all study participants. This will allow the research team to determine adherence with the SPIN-VR programme such as how often and for how long participants are using the system for each exercise.

Participants will be asked to do the exercise programme three times a week for 12 weeks at home. At the end of these 12 weeks, they will be asked to return for the first follow-up visit and to return the equipment. Patients can continue to perform the exercises they learnt without the SPIN-VR. To personalise the SPIN-VR games and set the optimal difficulty level for each participant, the system allows adjustments based on factors such as the number of obstacles, speed, repetitions, range of motion and dual-tasking complexity. The games included in the SPIN-VR replicate the following exercises:

1. Weight shifting (shifting weight in the hips).
2. Lunging/step forward (placing one leg forward and applying weight to it).
3. Stand up and sit down (standing upright from a seated position in a chair).
4. High stepping (lifting each knee up and down).

The games will be progressed during the 12 weeks if participants scored above a threshold on a composite total score so they can progress to more advanced tasks. On a weekly basis, the participant and physiotherapist can have access to a report on performance and number of times and for how long the participant has interacted with the system. The individual will continue to do the SPIN-VR programme and will have the option of scheduling up to two appointments during the 12 weeks with their physiotherapist.

### Comparator

The comparator is standard physiotherapy care that will consist of an initial assessment and follow-on face-to-face or remote follow-up sessions with the physiotherapist as is normally delivered within the physiotherapy service. Usual care treatment will be physiotherapy advice and exercise^48^ as decided by the treating physiotherapist.

### Recruitment

Participants will be identified by a member of the clinical physiotherapy team in outpatient clinics in the University Hospital of Wales in Cardiff. Patients from the waiting list for physiotherapy care, and those already attending musculoskeletal clinics, will be screened. They will be screened for eligibility against the eligibility criteria, and if they are interested, they will be offered a participant information sheet. Contact details will be obtained and passed on to the research team, who will then approach participants to arrange a baseline assessment and informed consent visit. A copy of the consent form is attached in the online supplemental material. Consent to share patient contact details with the research team will be obtained beforehand.

### Randomisation and blinding

Randomisation will be provided via a computer-generated list. The research team will access the web-based randomisation module and enter the required data and confirmation of patient eligibility and consent. Allocation is concealed until randomisation is complete and irreversible within the system. The researcher can then inform the participant of their allocation. Because of the nature of the intervention, patients and members of the research team will not be blinded to study allocation.

### Safety

The physical component of the intervention does not differ from what a patient would be expected to do in usual care physiotherapy. Therefore, it poses a minimal risk. For this study, serious AEs that are related to the intervention and unexpected events occurring from the time of randomisation until the participant’s 24-week follow-up will be recorded. AEs will be recorded through standard data collection.

### Sample size

This feasibility study aims to recruit and randomise 50 participants, a sample size chosen to provide adequate precision in estimating key feasibility parameters, specifically retention and adherence rates, with a 95% confidence intervals of approximately ±10%.^49 50^ As the primary objective is to evaluate feasibility rather than effectiveness, no formal power calculation was performed.^51^ The sample size was pragmatically determined based on an anticipated recruitment rate of approximately four participants per month over a 13-month period, with an estimated 60% of eligible patients expected to consent to participation. This conservative sample size is intended to generate essential data to inform the design and sample size calculations of a future pilot or definitive RCT.

### Data analysis

The main outcome of recruitment feasibility will be reported, as frequency of patients recruited during the study period. Reasons for screening or randomisation failure will be collated and presented as frequencies. Descriptive data will include an evaluation of eligibility, recruitment, acceptability of and adherence to the intervention, with 95% CIs. Secondary outcome measures, including PROMs, will be assessed before and after the intervention to establish any possible trend in the intervention effects over time and understand the variability in data. The completion of outcome measures will be reported and changes in outcome assessments relative to baseline assessments will be analysed using appropriate parametric or non-parametric statistics based on the characteristics of the data. All tests will be two-sided with an alpha level of 0.05. Estimates of population variances of outcomes for future power calculations will use the upper 80th percentile of confidence intervals around the estimates will be carried out. In addition, individual performance and movement quality in the VR intervention will be monitored and measured during the 12-week programme, every time the patient completes a training session, to determine any improvement in comparison to baseline measurements.

The qualitative interviews will be used to obtain views on participants’ acceptance of the technology, factors influencing their participation in the trial, any unexpected benefits or adverse effects experienced, as well as their experiences with pain and any impact on their overall function and exercise. Data will be managed using NVivo software. Data will be analysed using Braun and Clarke’s thematic analysis.

### Breaches of GCP or protocol

Protocol deviations are departures from the approved protocol. Prospective, planned deviations or waivers to the protocol must not be used, except to protect the rights, safety and well-being of human subjects under emergency circumstances. Accidental protocol deviations can happen at any time. Recurring deviations from the protocol are not acceptable, will require immediate action and could potentially be classified as a serious breach. Deviations must be documented on the relevant study form by the Principal Investigator (PI) or their representative and reported to the Chief Investigator (CI) and Centre for Healthcare Evaluation, Device Assessment, and Research (CEDAR) immediately. Deviations may also be identified during trial monitoring visits.

A ‘serious breach‘’ is a breach of Good Clinical Practice (GCP) or the protocol which is likely to affect to a significant degree:

1. The safety or physical or mental integrity of the participants of the trial.
2. The scientific value of the trial.

CEDAR will notify the sponsor immediately of any potentially serious breach. The incident will be investigated by the sponsor who will determine whether the breach constitutes a serious breach. CEDAR (on behalf of the sponsor) will report serious breaches to the local NHS research governance department and will inform the research ethics committee (REC) in writing within 7 days. Any corrective action required will be undertaken by the CI/CEDAR and REC and informed. If necessary, a protocol amendment will be submitted for review.

### Data protection and patient confidentiality

All investigators and study site staff must comply with the requirements of the General Data Protection Regulation and Data Protection Act 2018 with regard to the collection, storage, processing and disclosure of personal information and will uphold the act’s core principles.

The data custodian in this study is MA-A on behalf of the sponsor, and the data will be held on CU’s secure server.

Study case report Forms (CRFs) will be kept in secure locations (locked cupboard) at the study site and at CEDAR. The study database will be accessible only by delegated study personnel involved in the study.

### Amendments

It is the sponsor and CI’s responsibility to classify amendments as being non-substantial or substantial. On behalf of the sponsor and CI, CEDAR will obtain approval from the REC and Health and Care Research Wales/Health Care Authority (HCRW/HRA) for all substantial amendments to the original approved documents.

Amendments will not be implemented until all relevant regulatory organisations have granted a favourable opinion (or no objection), and local site research and development (R&D) office approval has been received.

## Ethics and dissemination

### Dissemination policy

The data will be analysed and tabulated and a final trial report will be prepared on completion of the trial by the CI. Full trial report can be accessed at the sponsor’s internal network. There are no time limits or review requirements on the publications arising from the trial. The Funder will be acknowledged in the publications arising from this trial. Participants of the trial will have a right to obtain publications arising from the trial, on formal request. Participants of the trial will have a right to obtain their individual results, on formal request and after the results have been published. Full trial report, anonymised participant-level dataset and statistical code for generating the results will be made publicly available.

###  Ethical approval

This protocol was approved by the Wales REC 3. (REC Reference: 23/WA/0311).

### Monitoring

A trial steering committee (TSC) has been established to provide oversight and support to the study. The committee comprises three independent members. An independent physiotherapy in a different health board who acts as the chair, an independent researcher in VR technology and a Patient and Public Involvement (PPI) member. The CI and the trial manager will also attend these meetings. A TSC terms of reference has been put in place to guide the TSC and meetings. The TSC will meet as required, and at least every 6 months.

A trial management group (TMG) has been established. This includes the CI, trial managers, research assistants and members of the physiotherapy team from Cardiff and Vale University Health Board. The TMG meetings will be held at least monthly to monitor all aspects of the study.

## Supplementary material

10.1136/bmjopen-2025-102994 online supplemental file 1

## References

[1] Cross M, Smith E, Hoy D (2014). The global burden of hip and knee osteoarthritis: estimates from the global burden of disease 2010 study. Ann Rheum Dis.

[2] Steinmetz JD, Culbreth GT, Haile LM (2023). Global, regional, and national burden of osteoarthritis, 1990–2020 and projections to 2050: a systematic analysis for the Global Burden of Disease Study 2021. Lancet Rheum.

[3] Safiri S, Kolahi A-A, Smith E (2020). Global, regional and national burden of osteoarthritis 1990-2017: a systematic analysis of the Global Burden of Disease Study 2017. Ann Rheum Dis.

[4] Mills K, Hunt MA, Leigh R (2013). A systematic review and meta-analysis of lower limb neuromuscular alterations associated with knee osteoarthritis during level walking. Clin Biomech (Bristol).

[5] Kim D-J (2019). A study on the physical activities, mental health, and health-related quality of life of osteoarthritis patients. Osong Public Health Res Perspect.

[6] Al-Ahaideb A, Alrushud A, El-Sobkey S (2013). Impact of knee osteoarthritis on the quality of life among Saudi elders: a comparative study. *Saudi J Sports Med*.

[7] Jordan KM, Arden NK, Doherty M (2003). EULAR Recommendations 2003: an evidence based approach to the management of knee osteoarthritis: report of a task force of the standing committee for international clinical studies including therapeutic trials (ESCISIT). Ann Rheum Dis.

[8] Button K, Nicholas K, Busse M (2018). Integrating self-management support for knee injuries into routine clinical practice: TRAK intervention design and delivery. Musculoskelet Sci Pract.

[9] Charlesworth J, Fitzpatrick J, Perera NKP (2019). Osteoarthritis- a systematic review of long-term safety implications for osteoarthritis of the knee. BMC Musculoskelet Disord.

[10] Fernandes L, Hagen KB, Bijlsma JWJ (2013). EULAR recommendations for the non-pharmacological core management of hip and knee osteoarthritis. Ann Rheum Dis.

[11] Jordan JL, Holden MA, Mason EE (2010). Interventions to improve adherence to exercise for chronic musculoskeletal pain in adults. Cochrane Database Syst Rev.

[12] Rice D, Nijs J, Kosek E (2019). Exercise-induced hypoalgesia in pain-free and chronic pain populations: state of the art and future directions. J Pain.

[13] Kami K, Tajima F, Senba E (2017). Exercise-induced hypoalgesia: potential mechanisms in animal models of neuropathic pain. Anat Sci Int.

[14] Naugle KM, Fillingim RB, Riley JL (2012). A meta-analytic review of the hypoalgesic effects of exercise. J Pain.

[15] Bandak E, Boesen M, Bliddal H (2015). Associations between muscle perfusion and symptoms in knee osteoarthritis: a cross sectional study. Osteoarthr Cartil.

[16] Bandak E, Boesen M, Bliddal H (2019). Exercise-induced pain changes associate with changes in muscle perfusion in knee osteoarthritis: exploratory outcome analyses of a randomised controlled trial. BMC Musculoskelet Disord.

[17] Lluch E, Torres R, Nijs J (2014). Evidence for central sensitization in patients with osteoarthritis pain: a systematic literature review. Eur J Pain.

[18] Lluch Girbés E, Nijs J, Torres-Cueco R (2013). Pain treatment for patients with osteoarthritis and central sensitization. Phys Ther.

[19] Hurley M, Dickson K, Hallett R (2018). Exercise interventions and patient beliefs for people with hip, knee or hip and knee osteoarthritis: a mixed methods review. Cochrane Database Syst Rev.

[20] Cisternas AF, Ramachandran R, Yaksh TL (2020). Unintended consequences of COVID-19 safety measures on patients with chronic knee pain forced to defer joint replacement surgery. Pain Rep.

[21] Papalia R, Vasta S, Tecame A (2013). Home-based vs supervised rehabilitation programs following knee surgery: a systematic review. Br Med Bull.

[22] Beard DJ, Dodd CAF (1998). Home or supervised rehabilitation following anterior cruciate ligament reconstruction: a randomized controlled trial. *J Orthop Sports Phys Ther*.

[23] Grant JA, Mohtadi NGH, Maitland ME (2005). Comparison of home versus physical therapy-supervised rehabilitation programs after anterior cruciate ligament reconstruction: a randomized clinical trial. Am J Sports Med.

[24] López-Liria R, Padilla-Góngora D, Catalan-Matamoros D (2015). Home-based versus hospital-based rehabilitation program after total knee replacement. Biomed Res Int.

[25] Jack K, McLean SM, Moffett JK (2010). Barriers to treatment adherence in physiotherapy outpatient clinics: a systematic review. Man Ther.

[26] Essery R, Geraghty AWA, Kirby S (2017). Predictors of adherence to home-based physical therapies: a systematic review. Disabil Rehabil.

[27] Lee F-K, Lee T-F, So W-W (2016). So, Effects of a tailor-made exercise program on exercise adherence and health outcomes in patients with knee osteoarthritis: a mixed-methods pilot study. Clin Interv Aging.

[28] Bachmann C, Oesch P, Bachmann S (2018). Recommendations for Improving Adherence to Home-Based Exercise: a Systematic Review. *Phys Med Rehab Kuror*.

[29] Massetti T, da Silva TD, Crocetta TB (2018). The clinical utility of virtual reality in neurorehabilitation: a systematic review. J Cent Nerv Syst Dis.

[30] Lei C, Sunzi K, Dai F (2019). Effects of virtual reality rehabilitation training on gait and balance in patients with Parkinson’s disease: a systematic review. PLoS One.

[31] Schiza E, Matsangidou M, Neokleous K (2019). Virtual reality applications for neurological disease: a review. Front Robot AI.

[32] Al-Amri M (2016). Augmented Feedback Approach to Double-Leg Squat Training for Patients with Knee Osteoarthritis: A Preliminary Study.

[33] Al-Amri M, Roos PE, Button K (2013). 2013 International Conference on Virtual Rehabilitation (ICVR).

[34] O’Cathain A, Croot L, Duncan E (2019). Guidance on how to develop complex interventions to improve health and healthcare. BMJ Open.

[35] A. RD (2000). Recommendations for the medical management of osteoarthritis of the hip and knee: 2000 update; American College of Rheumatology Subcommittee on Osteoarthritis Guidelines. Arthritis Rheum.

[36] Phillips D, Lamont T, Tinati T (2018). Moving Forward: Physiotherapy for Musculoskeletal Health and Wellbeing.

[37] Altman R, Asch E, Bloch D (1986). Development of criteria for the classification and reporting of osteoarthritis: classification of osteoarthritis of the knee. *Arthritis & Rheumatism*.

[38] Gill S, McBurney H (2008). Reliability of performance-based measures in people awaiting joint replacement surgery of the hip or knee. Physiother Res Int.

[39] Kennedy DM, Stratford PW, Wessel J (2005). Assessing stability and change of four performance measures: a longitudinal study evaluating outcome following total hip and knee arthroplasty. BMC Musculoskelet Disord.

[40] Stausholm MB, Bjordal JM, Moe-Nilssen R (2023). Pain pressure threshold algometry in knee osteoarthritis: intra- and inter-rater reliability. Physiother Theory Pract.

[41] Alghadir AH, Anwer S, Iqbal A (2018). Test-retest reliability, validity, and minimum detectable change of visual analog, numerical rating, and verbal rating scales for measurement of osteoarthritic knee pain. J Pain Res.

[42] Roos EM, Roos HP, Lohmander LS (1998). Knee Injury and Osteoarthritis Outcome Score (KOOS)—development of a self-administered outcome measure. *J Orthop Sports Phys Ther*.

[43] Group TE (1990). EuroQol - a new facility for the measurement of health-related quality of life. Health Policy.

[44] Beecham J, Knapp M (2001). Costing psychiatric interventions. Measuring Mental Health Needs.

[45] Craig CL, Marshall AL, Sjöström M (2003). International physical activity questionnaire: 12-country reliability and validity. Med Sci Sports Exerc.

[46] Lorig KR, Sobel DS, Ritter PL (2001). Effect of a self-management program on patients with chronic disease. Eff Clin Pract.

[47] Akin-Akinyosoye K, James RJE, McWilliams DF (2021). The Central Aspects of Pain in the Knee (CAP-Knee) questionnaire; a mixed-methods study of a self-report instrument for assessing central mechanisms in people with knee pain. Osteoarthr Cartil.

[48] Button K, van Deursen RW, Soldatova L (2013). TRAK ontology: defining standard care for the rehabilitation of knee conditions. J Biomed Inform.

[49] Sim J, Lewis M (2012). The size of a pilot study for a clinical trial should be calculated in relation to considerations of precision and efficiency. J Clin Epidemiol.

[50] Teare MD, Dimairo M, Shephard N (2014). Sample size requirements to estimate key design parameters from external pilot randomised controlled trials: a simulation study. Trials.

[51] Billingham SAM, Whitehead AL, Julious SA (2013). An audit of sample sizes for pilot and feasibility trials being undertaken in the United Kingdom registered in the United Kingdom Clinical Research Network database. BMC Med Res Methodol.

