# Supplementary material for: Evaluating home-based personalised virtual reality physiotherapy rehabilitation compared with usual care in the treatment of pain for people with knee osteoarthritis: protocol for a randomised feasibility study
Source: BMJ Open. 2025 Oct 21;15(10):e102994. doi: 10.1136/bmjopen-2025-102994 (PMC12548606; doi:10.1136/bmjopen-2025-102994)
Supplement: online supplemental file 1 [file bmjopen-15-10-s001.pdf]

SPIN-VR Informed Consent Form (ICF)

SHORT TITLE: Sensor-based physiotherapy intervention with virtual reality (SPIN-VR)

FULL TITLE: A randomised feasibility study to evaluate the home-based personalised virtual reality physiotherapy rehabilitation compared to usual care in the treatment of pain for people with knee osteoarthritis.

Chief Investigator: Dr Mohammad Al-Amri, Cardiff University

Participant Identification Number: __________

Please initial box

1. I confirm that I have read and understand the information sheet dated .... (version.........) for the above study. I have had the opportunity to consider the information, ask questions, and have had these answered satisfactorily. ☐

2. I understand that my participation is voluntary and that I am free to withdraw at any time without giving any reason, without my medical care or legal rights being affected. ☐

3. I understand that relevant sections of my medical records and data collected during the study may be looked at by responsible individuals from Cardiff University, Cardiff and Vale UHB, and other regulatory authorities where it is relevant to taking part in this research. ☐

4. I understand that data collected from this study will be shared anonymously with external collaborations In the UK and abroad, including commercial partners ☐

5. I consent to my movements, while performing exercises, being recorded using audio-visual equipment and that digital masking will be used to ensure anonymity of the footage is used in any publication or public and scientific presentations. ☐

6. I understand that all staff involved in this study must comply with the requirements of the General Data Protection Regulation (GDPR) and Data Protection Act 2018 with regards to the collection, storage, processing, and disclosure of personal information. ☐

7. I consent to my general practitioner (GP) being contacted in the event of unforeseen medical and/or psychological issues that are reported during the study. ☐

8. If I decide to withdraw, or am withdrawn from the study, I agree that the information collected about me up to the point of my withdrawal will be used in the data analysis for this study. ☐

9. I agree to take part in this research study. ☐

10. (OPTIONAL) I consent to being interviewed by a Cardiff University researcher, and to the interview being audio recoded. ☐

11. (OPTIONAL) I consent to any verbatim quotations I make during the interview being anonymously published in the study results. ☐

12. (OPTIONAL) I understand that my audio recording from the interview will use a pseudonymised code, and be sent to an approved third-party company to be transcribed. ☐

13. (OPTIONAL) I consent to being contacted at a later date and invited to take part in any future relevant research studies. ☐

______________________	______________________	______________________
Name of Participant	Date	Signature of Participant
______________________	______________________	______________________
Name of Researcher	Date	Signature of Researcher
