# Supplementary material for: Evaluating home-based personalised virtual reality physiotherapy rehabilitation compared with usual care in the treatment of pain for people with knee osteoarthritis: protocol for a randomised feasibility study
Source: BMJ Open. 2025 Oct 21;15(10):e102994. doi: 10.1136/bmjopen-2025-102994 (PMC12548606; doi:10.1136/bmjopen-2025-102994)
Supplement: Supplementary file 2 [file bmjopen-2025-102994.reviewer_comments.pdf]

PEER REVIEW HISTORY

BMJ Open publishes all reviews undertaken for accepted manuscripts. Reviewers are asked to complete a checklist review form and are provided with free text boxes to elaborate on their assessment. These free text comments are reproduced below.

ARTICLE DETAILS

Title (Provisional)

Protocol for a randomised feasibility study to evaluate home-based personalised virtual reality physiotherapy rehabilitation compared to usual care in the treatment of pain for people with knee osteoarthritis.

Authors

Al-Amri, Mohammad; Bird, Samuel; Nistor, Denise-Teodora; White, Judith; Button, Kate; Warner, Martin; Walsh, David; Shorten, Dione; Evans, Rose

---

VERSION 1 - REVIEW

**Reviewer**	**1**
**Name**	**Rasova, Kamila**
**Affiliation**	**Charles University Third Faculty of Medicine, Department of Rheumatology and Rehabilitation**
**Date**	**18-Jun-2025**
**COI**	**None**

Overall, I find the protocol very well prepared and comprehensive. I would, however, recommend addressing the following minor points to further enhance clarity and completeness:

1. Study Design Section

Please consider including the specific assessments that will be conducted as part of the study. This would provide a clearer picture of the methodological framework.

2. Sample Size Estimation

It would be helpful to include information on how the estimated sample size was calculated, including assumptions (e.g. effect size, power, significance level).

3. Section 7.5 – Trial Assessments

o Please add references for each assessment tool used.

o Indicate which values represent better outcomes (e.g. higher scores, shorter times, greater ranges).

4. Description of VR Exercises

A more detailed explanation of the exercises to be performed in virtual reality would be appreciated. Ideally, this could be accompanied by an illustrative figure or screenshot to facilitate understanding.

5. Biomechanically Validated Body-Worn Sensors

o Please specify the type(s) of sensors to be used, including model and manufacturer if available.

o Clarify how many sensors will be used and where on the body they will be placed.

6. Exercise Frequency

The paper notes that participants will be asked to perform the exercise program three times per week. It may be worth considering:

o How likely it is that some participants will wish to exceed this frequency.

o How such cases will be addressed within the study protocol (e.g. permitted, recorded, restricted?).

7. Interaction Monitoring

While the number and duration of interactions with the system are to be reported to the physiotherapist weekly, could additional metrics be included? For example, more detailed data on the actual movements performed (e.g. repetitions, range of motion) could provide valuable insights into adherence and performance.

8. Movement Quality Monitoring in VR

The paper mentions that movement quality will be monitored.

o Will this be done through automatic system evaluation?

o If so, please clarify how movement quality will be assessed (e.g. algorithmic scoring, comparison to normative patterns).

9. System Capabilities

It would be valuable to include more detail on the technical capabilities of the VR system. For example, does the system allow for:

o Estimation of squat depth

o Maximum joint angles and ranges

o Movement symmetry

o Postural sway tracking?

VERSION 1 - AUTHOR RESPONSE

Comment number	Reviewer's comment	Author's response
6	Please consider including the specific assessments that will be conducted as part of the study. This would provide a clearer picture of the methodological framework.	More specific information given regarding the non-PROM assessments that are conducted during the study.
7	It would be helpful to include information on how the estimated sample size was calculated, including assumptions (e.g. effect size, power, significance level).	The proposed sample size of 50 participants is in line with established methodological guidance for feasibility studies, where the primary aim is not to assess intervention effectiveness, but to estimate key parameters needed to inform the design of a future definitive trial (e.g. retention, adherence, and acceptability).<br>Specifically, a sample of 50 allows us to estimate a retention or adherence rate with a 95% confidence interval of ±10%, which is considered sufficiently precise for feasibility purposes (Teare et al., 2014; Sim & Lewis, 2012; Billingham et al., 2013). This level of precision will help determine whether progression to a full trial is justified and feasible and inform appropriate sample size calculations for that phase.<br>Also, this sample size supports meaningful exploration of recruitment procedures, data collection processes, and intervention delivery in real-world conditions, which are key objectives of feasibility work.<br>We have clarified this rationale in the revised manuscript to better reflect the purpose and justification of the sample size.
8	Please add references for each assessment tool used.	References added for 30 second time sit to stand test, timed 6 metre walk test, Pressure Pain Detection Thresholds, and Self-report measures of pain mechanisms.
9	Indicate which values represent better outcomes (e.g. higher scores, shorter times, greater ranges).	Added in descriptions of what values equal in terms of outcomes where relevant in the "Trial assessments" section.

10	A more detailed explanation of the exercises to be performed in virtual reality would be appreciated. Ideally, this could be accompanied by an illustrative figure or screenshot to facilitate understanding.	A description of each of the exercises in the VR has been added in under the ‘Intervention’ section.
11	Please specify the type(s) of sensors to be used, including model and manufacturer if available.	The description of the sensor configuration has been clarified to state that the VRHP system uses five biomechanically validated Movella DOT inertial measurement units (IMUs).
12	Clarify how many sensors will be used and where on the body they will be placed.	Also clarified by stating that “positioned on the upper and lower limbs in accordance with the movement requirements of each virtual exercise. Sensor placement is task-specific to ensure accurate motion capture and feedback during exercise performance”
13	The paper notes that participants will be asked to perform the exercise program three times per week. It may be worth considering:<br>• How likely it is that some participants will wish to exceed this frequency.<br>How such cases will be addressed within the study protocol (e.g. permitted, recorded, restricted?).	Participants are required to return the equipment at the end of the 12 weeks. They are told this. However, the exercises they learn from the VRHP can be performed without the games, and patients are told they can continue with these exercises if they would like to. This has been added to the “Intervention” section.
14	While the number and duration of interactions with the system are to be reported to the physiotherapist weekly, could additional metrics be included? For example, more detailed data on the actual movements performed (e.g. repetitions, range of motion) could provide valuable insights into adherence and performance.	This is not being performed in the study and so nothing has been added. However, these comments are useful and will be considered for future research.
15	The paper mentions that movement quality will be monitored:<br>• Will this be done through automatic system evaluation?<br>If so, please clarify how movement quality will be assessed (e.g. algorithmic scoring, comparison to normative patterns).	Thank you for your constructive comments. We have now clarified this aspect in the revised manuscript.<br><br>The system incorporates an automatic personalisation mechanism that adjusts key gameplay parameters, including difficulty and speed, in real time based on user performance. Movement quality is continuously monitored through parameters such

		as joint angle attainment, which are selected according to the therapeutic goals associated with each specific game.<br><br>To enable meaningful and individualised assessment, each session begins with a baseline assessment trial. This trial is used to define personalised thresholds for key biomechanical parameters (e.g., joint ranges, movement targets), which are then used to inform both real-time game adaptation and subsequent offline analysis.<br><br>During gameplay, the system utilises real-time data captured from IMUs to evaluate whether users are meeting predefined movement targets and demonstrating progression (e.g., through improved scoring and consistency). Based on these real-time evaluations, the game dynamically adapts to maintain an appropriate level of challenge.
16	It would be valuable to include more detail on the technical capabilities of the VR system. For example, does the system allow for:<br>• Estimation of squat depth<br>• Maximum joint angles and ranges<br>• Movement symmetry<br>• Postural sway tracking	Further to our previous response, in addition to real-time monitoring, kinematic data are continuously captured and stored for offline analysis. While certain parameters may also be utilised in real time, depending on the specific requirements and objectives of the game, offline analysis allows for a more comprehensive evaluation of movement quality. This includes:<br><br>• Estimation of squat depth<br>• Calculation of maximum joint angles and joint range of motion<br>• Analysis of movement symmetry<br>• (Potentially) tracking of postural sway, depending on the sensor configuration<br><br>These offline assessments facilitate longitudinal tracking of user performance and support data-driven therapeutic decision-making. The manuscript has been revised to

		explicitly detail these technical capabilities and their contributions to both real-time game adaptation and post-session evaluation.

VERSION 2 - REVIEW

Reviewer **1**

Name **Rasova, Kamila**

Affiliation **Charles University Third Faculty of Medicine, Department of Rheumatology and Rehabilitation**

Date **18-Sep-2025**

COI

The authors have addressed all comments thoroughly, and the manuscript has been substantially improved. The text is now clear, well-structured, and methodologically sound. The introduction provides a strong rationale and the study design is appropriately justified. Congratulation!
